# Multi-cohort meta-analysis, single-cell and Mendelian randomization analyses with experimental validation identify RHBDF2 as a shared mediator of ulcerative colitis and blood–brain barrier disruption

**DOI:** 10.3389/fnsyn.2026.1970578

**Published:** 2026-10-07

**Authors:** Jiongjiong Cheng, Aman Xu

**Affiliations:** 1 Department of General Surgery, The First Affiliated Hospital of Anhui Medical University, Hefei, Anhui, China; 2 Department of Emergency Surgery, The First Affiliated Hospital of Anhui Medical University, Hefei, Anhui, China

**Keywords:** ADAM17, blood–brain barrier, GW280264X, Mendelian randomization, meta-analysis, RHBDF2/iRhom2, single-cell RNA sequencing, TNFα

## Abstract

**Background:**

Ulcerative colitis (UC) is increasingly associated with central nervous system comorbidities, including cognitive decline and dementia, yet the molecular mechanisms linking intestinal inflammation to brain dysfunction remain unclear. Blood–brain barrier (BBB) disruption is a plausible convergence point, but the mediators involved are largely unknown.

**Methods:**

We performed random-effects meta-analyses of five human UC bulk-transcriptome cohorts (1,130 UC and 508 control samples) and three mouse BBB-disruption (BBBD) cohorts, followed by cross-species orthologue mapping to identify shared genes. The cellular context of candidate genes was resolved by single-cell RNA-sequencing analysis of human UC colonic mucosa (GSE214695) and paired human BBB-intact/BBB-opened brain samples (GSE208074), combined with scTenifoldKnk in-silico gene knockout. Summary-data-based Mendelian randomization (SMR) integrating whole-blood cis-eQTL and FinnGen GWAS data was used to assess causal relevance to dementia. Findings were validated in dextran sulfate sodium (DSS)-induced colitis in mice preventively treated with the ADAM17 inhibitor GW280264X, with Evans blue assessment of BBB permeability.

**Results:**

Four genes—TNF, RHBDF2, PILRA and CXCL16—were significantly and concordantly up-regulated in both UC and BBBD, forming a protein–protein association module organized around the sheddase ADAM17. RHBDF2, the essential activating chaperone of ADAM17, was predominantly up-regulated in colonic epithelial cells in UC, whereas its strongest inferred regulatory impact at the human BBB mapped to endothelial cells, affecting complement, microglial and angiogenic programs. Genetically predicted higher expression of RHBDF2 and PILRA was associated with increased risk of all-cause and Alzheimer’s dementia. In DSS-induced colitis, preventive administration of GW280264X attenuated intestinal inflammation, reduced cerebral Evans blue extravasation and lowered brain Rhbdf2, Adam17 and Tnfα protein levels.

**Conclusion:**

Integrative transcriptomic, genetic and experimental evidence identifies an RHBDF2–ADAM17–TNFα axis as a plausible mechanistic bridge between ulcerative colitis and blood–brain barrier disruption and a candidate therapeutic target.

## 1 Introduction

Ulcerative colitis (UC) is a chronic, relapsing inflammatory disorder of the colonic mucosa and, together with Crohn’s disease, constitutes the two major forms of inflammatory bowel disease (IBD). Over the past three decades, IBD has evolved into a global disease: although incidence has plateaued at a high level in Western nations, newly industrialized countries are experiencing a rapidly accelerating rise in incidence, and the worldwide prevalence continues to grow (Ng et al., 2017; Dharni et al., 2024). UC is increasingly recognized as a systemic disorder rather than a disease confined to the gut. Extraintestinal manifestations involving the joints, skin, eyes and hepatobiliary tract affect approximately one quarter of patients (Kilic et al., 2024; Larsen et al., 2010), and accumulating clinical and experimental evidence indicates that the central nervous system (CNS) is likewise affected.

Patients with IBD carry a substantially increased burden of psychiatric comorbidity, with pooled prevalences of anxiety and depressive symptoms of roughly one third and one quarter, respectively (Massironi et al., 2025; Barberio et al., 2021). Multi-omics profiling of patients with active UC has linked depression and anxiety to gut dysbiosis and disturbed serum metabolism (Yuan et al., 2021); notably, transfer of colitis-associated microbiota into germ-free mice induces anxiety-like behavior even in the absence of intestinal or neuroinflammation (Vicentini et al., 2022), and single-nucleus transcriptomics has identified hippocampal neuronal subpopulations that may mediate these behavioral changes (Luo et al., 2026). Beyond affective disorders, epidemiological evidence now connects IBD with neurodegeneration. A nationwide Taiwanese cohort study found that patients with IBD developed dementia more than twice as often as matched controls and were diagnosed approximately 7 years earlier (Zhang et al., 2021); a meta-analysis of seven studies confirmed an elevated risk of dementia in IBD (risk ratio 1.35), with an even stronger association for Alzheimer’s disease (Zhang et al., 2022); and a Danish population-based study reported a modest but significant increase in all-cause dementia among patients with UC (Rønnow Sand et al., 2022). These observations have given rise to the concept of “enterogenic dementia,” in which gut-derived inflammation promotes cognitive decline, although the underlying mechanisms remain poorly defined (Alshammari et al., 2025).

One plausible mechanistic link between intestinal inflammation and CNS dysfunction is the blood–brain barrier (BBB). Experimental colitis increases BBB permeability to small molecules in both rabbits and rats, accompanied by reduced expression of endothelial barrier antigen, independent of changes in cerebral blood flow (Hathaway et al., 1999; Natah et al., 2005). Intestinal inflammation also remodels a vascular barrier in the choroid plexus through bacteria-derived lipopolysaccharide, and genetically driven closure of this barrier produces short-term memory deficits and anxiety-like behavior, supporting a deregulated gut–brain vascular axis in IBD-related mental symptoms (Carloni et al., 2021; Carloni and Rescigno, 2022). Barrier disruption during colitis is not restricted to the brain: the blood–myenteric barrier of the enteric nervous system likewise degrades in a macrophage-dependent manner (Dora et al., 2021). In a human *in vitro* BBB model, plasma from patients with IBD and tumor necrosis factor α (TNFα) itself increased endothelial permeability, tight-junction disarrangement and monocyte transmigration—effects reversed by anti-inflammatory treatment (da Rocha et al., 2022)—whereas restoration of both intestinal and blood–brain barrier integrity by probiotic-derived extracellular vesicles rescued colitis-associated cognitive impairment in mice (Chen et al., 2026). Collectively, these findings position the BBB as a convergence point between peripheral intestinal inflammation and brain dysfunction, yet the molecular mediators remain largely unknown.

The clinical significance of BBB disruption is underscored by its established role in cognitive disorders. BBB breakdown is an early event in mild cognitive impairment and Alzheimer’s disease, with increased permeability to small molecules preceding or accompanying biomarker-defined pathology (Lin et al., 2021; Sweeney et al., 2019). In vascular mild cognitive impairment, greater BBB leakage on dynamic contrast-enhanced MRI correlates with worse cognitive performance (Li et al., 2021), and BBB permeability predicts white-matter injury and, indirectly, cognitive decline in older adults with and without neurodegenerative disease (Hergert et al., 2024). Mechanistically, disruption of endothelial–pericyte crosstalk within the neurovascular unit is a major driver of barrier failure in dementia (Procter et al., 2021), APOE ε4 carriers exhibit widespread BBB breakdown even before amyloid deposition or cognitive symptoms (Reas et al., 2024), and the hippocampus—a region central to the neuropsychiatric comorbidities of IBD—appears particularly vulnerable to barrier dysfunction (Davidson and Stevenson, 2024). Thus, identifying the molecules by which colitis compromises the BBB may reveal shared, druggable nodes linking UC to brain disorders.

Rhomboid 5 homolog 2 (RHBDF2; also known as inactive rhomboid protein 2, iRhom2) is a compelling candidate. RHBDF2 is a catalytically inactive pseudoprotease that acts as an essential cofactor for the maturation and substrate-selective activation of a disintegrin and metalloproteinase 17 (ADAM17), particularly in immune cells (Issuree et al., 2013; Maretzky et al., 2013). In the intestine, iRhom2 is up-regulated in the colonic mucosa of patients with IBD and in experimental colitis, where it promotes TNFα release and disease severity, whereas iRhom2 deficiency ameliorates colitis (Kim et al., 2020); conversely, combined loss of Rhbdf2 and Il10 precipitates spontaneous early-onset colitis, highlighting a context-dependent role in epithelial homeostasis (Geesala et al., 2019). Inflammatory cues such as TNFα plus interferon-γ transcriptionally induce iRhom2 in colon epithelial cells, enhancing ADAM17 maturation and the shedding of surface mediators and junctional adhesion molecules (Giese et al., 2021), and signaling through the iRhom2 cytoplasmic domain finely controls ADAM17 activation (Lu et al., 2025).

ADAM17 (TNFα-converting enzyme, TACE), the effector protease of this axis, is the principal sheddase of TNFα and its receptors. TACE activity is elevated in the colonic mucosa of patients with active UC (Brynskov et al., 2002) and rises during the induction phase of dextran sulfate sodium (DSS) colitis, while selective TACE inhibition attenuates weight loss, disease activity and cytokine production (Sharma et al., 2014); cell-specific deletion studies confirm ADAM17 as the primary sheddase controlling TNF and TNFR1 levels in inflammation (McMahan et al., 2013). Importantly, ADAM17 also acts directly on the cerebrovascular endothelium: it drives S100B/RAGE-dependent shedding of the endothelial glycocalyx after traumatic brain injury (Zou et al., 2022), mediates cytokine-induced cleavage of endomucin from the endothelial surface (Yang et al., 2020), and regulates survival signaling in brain microvascular endothelial cells (Wang et al., 2018). Pharmacological blockade of this pathway is feasible: GW280264X, an ADAM17 inhibitor, attenuates lipopolysaccharide-induced acute organ injury (Huner Yigit et al., 2025) and ameliorates psoriasis-like and endotoxin-induced inflammation in mice (Yu et al., 2025). Other molecules likewise bridge intestinal and CNS inflammation—the chemokine CXCL16 is up-regulated in the inflamed colon of patients with IBD and drives colonic inflammation (Diegelmann et al., 2010; Uza et al., 2011; Kalliomäki et al., 2014) while also promoting microglial activation and neuroinflammation in the brain (Dingyi et al., 2024), and the microglial receptor PILRA genetically modifies Alzheimer’s disease risk and microglial immunometabolism (Weerakkody et al., 2025; Lopatko Lindman et al., 2022). Nevertheless, a systematic, cross-species and cell-type-resolved map of the molecular connections between UC and BBB disruption is still lacking.

Summary-data-based Mendelian randomization (SMR) and related approaches that integrate expression quantitative trait loci (eQTL) with genome-wide association study (GWAS) data have proven powerful for prioritizing causal genes in both dementia and IBD (Liu et al., 2022; Wang et al., 2025; Li et al., 2025; Zhao et al., 2025), offering genetic anchoring that complements transcriptomic discovery. In the present study, we therefore combined (i) random-effects meta-analysis of five human UC bulk-transcriptome cohorts and three mouse BBB-disruption cohorts, (ii) cross-species orthologue mapping and integrative filtering, (iii) single-cell transcriptomic analyses and in-silico RHBDF2 knockout in human UC colon and human brain BBB cell types, (iv) SMR analysis of whole-blood cis-eQTL and disease GWAS data, and (v) *in vivo* validation in DSS-induced colitis with the ADAM17 inhibitor GW280264X. Through this integrative strategy, we identified RHBDF2 as a shared, cell-type-specific regulator common to UC and BBB disruption and tested the hypothesis that the RHBDF2–ADAM17–TNFα axis mediates colitis-associated blood–brain barrier injury.

## 2 Materials and methods

### 2.1 Study design and data sources

Five human ulcerative colitis (UC) bulk-transcriptome datasets (GSE193677, GSE206285, GSE224758, GSE235236 and GSE243625) and three mouse blood–brain barrier disruption (BBBD) datasets (GSE172102, GSE230212 and GSE248275) were obtained from the Gene Expression Omnibus (GEO);^1^ dataset details, platforms, group definitions and sample sizes are summarized in Table 1. Single-cell RNA-sequencing (scRNA-seq) data of colonic mucosa from six UC patients and six healthy controls (GSE214695) and paired sonicated/non-sonicated peritumoral brain biopsies from six recurrent glioblastoma patients undergoing low-intensity pulsed ultrasound (LIPU)-mediated BBB opening (GSE208074) were likewise retrieved from GEO. For summary-data-based Mendelian randomization (SMR), whole-blood cis-eQTL summary statistics were obtained from eQTLGen,^2^ and GWAS summary statistics for all-cause dementia, Alzheimer’s dementia, Parkinson’s dementia and vascular dementia were obtained from the FinnGen study.^3^

**Table 1:** Gene expression omnibus (GEO) datasets included in the meta-analyses.

Accession	Species	Platform	Disease model/cohort	Comparison	*n* (disease/control)	Reference (PMID)
GSE193677	*Homo sapiens*	GPL16791 Illumina HiSeq 2,500	MSCCR adult IBD cohort, colonic biopsy RNA-seq	UC vs. non-IBD control	872/461	36109152
GSE206285	*Homo sapiens*	GPL13158 Affymetrix HT HG-U133+ PM	UNIFI phase 3 trial baseline colonic biopsies	UC vs. healthy	186/18	36192482
GSE224758	*Homo sapiens*	GPL16791 Illumina HiSeq 2,500	Rectosigmoid colon biopsies	active UC vs. healthy	16/6	—
GSE235236	*Homo sapiens*	GPL21290 Illumina HiSeq 3,000	Colonic mucosa bulk RNA-seq	UC vs. non-IBD control	26/8	37495570
GSE243625	*Homo sapiens*	GPL16791 Illumina HiSeq 2,500	Colonic mucosa RNA-seq	UC (active + inactive) vs. healthy	30/15	39926325
GSE172102	*Mus musculus*	GPL21626 NextSeq 550	Cerebrovascular injury + LCMV infection (cerebral cortex)	CVI + LCMV vs. uninjured	4/4	34556874
GSE230212	*Mus musculus*	GPL24247 Illumina NovaSeq 6,000	*Cryptococcus neoformans* meningoencephalitis (brain)	Infected (H99 + cap59) vs. naive	6/3	40038673
GSE248275	*Mus musculus*	GPL24247 Illumina NovaSeq 6,000	LPS-induced sepsis-associated encephalopathy (cortical astrocyte RiboTag)	LPS 6 h/24 h vs. 0 h	12/6	39068155

### 2.2 Differential expression and random-effects meta-analysis

For each dataset, differential expression statistics (log2 fold change and its standard error, SE) for the disease-versus-control comparison were computed with limma (linear models with empirical Bayes moderation). Effect sizes were then combined across cohorts using random-effects meta-analysis implemented in the R package metafor, with restricted maximum-likelihood (REML) estimation of the between-study variance (tau^2^). Between-study heterogeneity was quantified with Cochran’s Q test and the I^2^ statistic, and meta-analytic *p* values were adjusted for multiple testing using the Benjamini–Hochberg (BH) method. A gene was retained as a robust meta-analytic differentially expressed gene (DEG) only when it (i) was measured in all cohorts (k = 5 for UC; k = 3 for BBBD), (ii) had |log2FC| > 0.585 and BH-adjusted FDR < 0.05, (iii) showed a consistent direction of change in every cohort (Sign_Consistent), and (iv) had a standard error smaller than the absolute effect size (SE < |β|).

### 2.3 Cross-species orthologue mapping and integrative filtering

Mouse BBBD DEGs were mapped to human orthologues using the HomoloGene database via the R package homologene; many-to-one mappings were collapsed to unique human genes. The human-mapped BBBD signature was intersected with the UC DEG signature, and shared genes were subjected to a three-layer direction-consistency filter requiring concordant directionality (i) within the BBBD cohorts, (ii) within the UC cohorts, and (iii) across the two diseases.

### 2.4 Functional enrichment and protein–protein association network analysis

Functional enrichment analysis was performed with Metascape^4^ against Gene Ontology Biological Process, KEGG, Reactome, WikiPathways and MSigDB gene-set collections, and the top 20 terms ranked by −log10(*p* value) were reported. Protein–protein association networks of the shared genes were constructed with STRING^5^ using the full evidence-channel view (curated databases, experimental interactions, gene neighborhood, gene fusions, gene co-occurrence, text mining, co-expression and protein homology). Because most shared genes are mechanistically linked to the ADAM17 sheddase pathway, ADAM17 was included as a bridging node. For the endothelial RHBDF2-regulated gene set, interaction networks were additionally constructed with GeneMANIA^6^.

### 2.5 Single-cell RNA-seq analysis

scRNA-seq data were processed with the Seurat R package (v5). After quality control (removal of low-quality cells and doublets based on detected gene/UMI counts and mitochondrial fraction), data were normalized, integrated across samples, and subjected to principal-component analysis, graph-based clustering and UMAP dimensionality reduction. Clusters were annotated into broad cell lineages using canonical marker genes—ten lineages in GSE214695 (epithelial, stromal, endothelial, B, plasma, CD4 T, CD8 T, other T, NK & ILC and myeloid cells) and eleven lineages in GSE208074. Cell-type proportions were computed per sample and for pooled groups. Gene expression differences at the single-cell level were assessed with the two-sided Wilcoxon rank-sum test with BH adjustment; sample-level comparisons (*n* = 6 per group) were performed with the unpaired two-tailed Student’s t-test. RHBDF2 protein localization in normal human colon was referenced from immunohistochemistry data of the Human Protein Atlas.^7^

### 2.6 In-silico gene knockout with scTenifoldKnk

Cell-type-specific gene-regulatory networks were constructed and virtual RHBDF2 knockout was performed using the scTenifoldKnk algorithm, which compares the regulatory network before and after in-silico deletion of the target gene via principal-component-based manifold alignment. In GSE214695, knockout was performed in epithelial cells (*n* = 6,575 cells from 6 HC and 6 UC samples); in GSE208074, to model RHBDF2 loss in an intact blood–brain barrier, knockout was performed using only cells from the non-sonicated (BBB-intact) peritumoral samples, separately in astrocytes (*n* = 786), endothelial cells (*n* = 1,085) and pericytes (*n* = 2,106), yielding gene-regulatory networks of 2,699, 2,728 and 2,785 genes, respectively. Perturbed genes were ranked by fold change (FC; a network-distance-based metric of regulatory rewiring, not an expression fold change) and Z score, with significance defined at *p* < 0.05 (BH-adjusted FDR reported); RHBDF2 itself was excluded from the perturbed-gene counts. Significantly perturbed genes were subjected to Metascape enrichment analysis and, for the endothelial gene set, GeneMANIA network analysis.

### 2.7 Summary-data-based Mendelian randomization (SMR) analysis

SMR analysis was performed using SMR v1.3.1 (Zhu et al., 2016) with the 1,000 Genomes European reference panel for LD estimation. Whole-blood cis-eQTL summary statistics from eQTLGen (2019 full release, SMR binary format; MAF ≥ 0.01) and FinnGen Release 10 GWAS summary statistics for four dementia endpoints—all-cause dementia (F5_DEMENTIA), Alzheimer’s dementia (F5_ALZHDEMENT), Parkinson’s dementia (PD_DEMENTIA_EXMORE) and vascular dementia (F5_VASCDEM)—were analyzed. Whole-blood cis-eQTL summary statistics from eQTLGen were converted into SMR .ma format (from VCF/PLINK files where necessary), and the top cis-eQTL of each of the four shared genes (TNF, RHBDF2, PILRA and CXCL16) was used as the instrumental variable. FinnGen GWAS summary statistics for four dementia endpoints were analyzed, and the heterogeneity in dependent instruments (HEIDI) test (20 SNPs per locus) was used to distinguish pleiotropy from linkage, with P_HEIDI ≥ 0.05 considered consistent with a shared causal variant. Within each phenotype, SMR *p* values across the four genes were adjusted using the BH method, and P_BH < 0.05 was considered significant. SMR effect-size plots were generated with the SMR plotting tool.

### 2.8 Animals and DSS-induced colitis model

Male C57BL/6 J mice (5 weeks old) were randomly allocated to three groups (*n* = 6 per group): normal control (NC, regular drinking water), model (Model, 2.5% (w/v) dextran sulfate sodium (DSS, MW 35,000–45,000; MedChemExpress) in drinking water for 7 consecutive days) and treatment [Treat, 2.5% DSS for 7 days plus GW280264X, an ADAM17 inhibitor (500 μg/kg, intraperitoneal injection once daily from the first day of DSS exposure, i.e., a preventive regimen) (Huner Yigit et al., 2025; Yu et al., 2025)]. Body weight was recorded daily, and the disease activity index (DAI) was scored daily as the mean of the three subscores for body-weight loss, stool consistency and occult/gross rectal bleeding (each 0–4). At the end of the experiment, mice were anesthetized by inhalation of isoflurane delivered with a calibrated vaporizer (induction at 3–4% in 100% oxygen in an induction chamber, followed by maintenance at 1.5–2% via a nose cone), and an adequate depth of anesthesia was confirmed by loss of the righting reflex and absence of the pedal withdrawal (toe-pinch) reflex. Euthanasia was then performed by exsanguination via transcardial perfusion with ice-cold saline under deep isoflurane anesthesia, and death was confirmed by cessation of heartbeat and respiration. Animals were monitored daily, and predefined humane endpoints were applied: any mouse showing body-weight loss exceeding 20% of baseline, persistent severe lethargy, or signs of unrelieved distress was to be euthanized immediately by the same procedure. All animal procedures were approved by the institutional animal care and use committee of the First Affiliated Hospital of Anhui Medical University.

### 2.9 Evans blue assessment of blood–brain barrier permeability

Blood–brain barrier permeability was assessed by Evans blue extravasation. Before tissue harvesting, all mice received a tail-vein injection of 2% (w/v) Evans blue (Sigma-Aldrich) in sterile saline at 4 mL/kg, and the dye was allowed to circulate for 1 h. At the end of the circulation period, mice were deeply anesthetized with isoflurane as described above and euthanized by exsanguination via transcardial perfusion with ice-cold saline until the effluent ran clear; this procedure both removed intravascular dye and constituted the method of euthanasia. Colons were photographed and their lengths measured; brains were photographed to document gross dye extravasation, weighed and homogenized, and the extravasated dye was extracted. The absorbance of the extract was measured spectrophotometrically at 620 nm, normalized to brain weight, and expressed relative to the NC group.

### 2.10 Reverse-transcription quantitative PCR (RT-qPCR)

Total RNA was extracted from colon tissue with TRIzol reagent, reverse-transcribed into cDNA, and amplified by quantitative PCR using SYBR Green chemistry. Relative mRNA levels of Tnf, Il6, Il1b and Rhbdf2 were calculated using the 2^−ΔΔCt method with Gapdh as the endogenous reference gene. Primer sequences are listed in Supplementary Table S1.

### 2.11 Western blotting

Brain tissue was lysed in RIPA buffer supplemented with protease and phosphatase inhibitors, and protein concentrations were determined by BCA assay. Equal amounts of protein were separated by SDS-PAGE and transferred to PVDF membranes. Membranes were incubated with primary antibodies against Rhbdf2 (polyclonal, Cat No. 23181-1-AP, 1:2,000), Adam17 (polyclonal, Cat No. 29948-1-AP, 1:2,000), Tnfα (polyclonal, Cat No. 17590-1-AP, 1:1,000) and Gapdh (monoclonal, clone 1E6D9, Cat No. 60004-1-Ig, 1:50,000; all from Proteintech), followed by HRP-conjugated secondary antibodies, and signals were developed with enhanced chemiluminescence.

### 2.12 Statistical analysis

Experimental data are presented as mean ± SEM. Body-weight and DAI time courses were analyzed by two-way repeated-measures ANOVA followed by Tukey’s multiple comparisons test; three-group endpoint comparisons (RT-qPCR, colon length, Evans blue content) were analyzed by one-way ANOVA followed by Tukey’s multiple comparisons test; and two-group sample-level comparisons were analyzed by unpaired two-tailed Student’s t-test. Analyses were performed with R (v4.5.3) and GraphPad Prism (v9.5), and *p* < 0.05 was considered statistically significant. Statistical procedures specific to the bioinformatic analyses (BH false-discovery-rate control, Wilcoxon rank-sum tests, HEIDI test) are described in the corresponding sections above.

## 3 Results

### 3.1 Multi-cohort meta-analysis identifies robust transcriptomic signatures of ulcerative colitis and blood–brain barrier disruption

To define the transcriptional alterations shared between ulcerative colitis (UC) and blood–brain barrier disruption (BBBD), we first performed independent random-effects meta-analyses across five human UC cohorts (GSE193677, GSE206285, GSE224758, GSE235236 and GSE243625; 1,130 UC and 508 healthy control samples in total) and three independent mouse BBBD cohorts (GSE172102, GSE230212 and GSE248275), which collectively model BBBD caused by cerebrovascular injury with subsequent viral infection, cryptococcal meningoencephalitis, and lipopolysaccharide (LPS)-induced systemic inflammation (sepsis-associated encephalopathy), respectively (Table 1). For each dataset, log2 fold changes (log2FC) and standard errors (SE) of disease-versus-control comparisons were combined using the *metafor* random-effects model. Genes were retained as robust differentially expressed genes (DEGs) only when they were measured in all cohorts (k = 5 for UC; k = 3 for BBBD), showed |log2FC| > 0.585 with Benjamini–Hochberg-adjusted FDR < 0.05, a consistent direction of change across cohorts, and a standard error smaller than the absolute effect size (SE < |β|). This stringent meta-analytic framework yielded 245 robust DEGs in UC and 141 robust DEGs in BBBD, all of which were up-regulated in the BBBD brain, indicating a stereotyped pro-inflammatory transcriptional response of the injured neurovascular unit.

### 3.2 Functional enrichment of UC and BBBD meta-signatures

Functional enrichment analysis of the two meta-signatures was performed with Metascape (Figures 1A,B). The 245 UC DEGs were predominantly enriched in processes related to epithelial barrier integrity, transport and metabolism, including cell junction organization (GO:0034330), organic anion transport (GO:0015711), carboxylic acid metabolic process (GO:0019752), cell adhesion molecule (CAM) interactions (hsa04514), cytokine–cytokine receptor interaction (hsa04060), extracellular matrix organization (R-HSA-1474244) and cell–substrate adhesion (GO:0031589), together with leukocyte activation, consistent with the dual barrier-disruption and immune-activation pathology of the UC mucosa (Figure 1A). In sharp contrast, the 141 BBBD DEGs were overwhelmingly enriched in innate immune and inflammatory pathways, led by inflammatory response (GO:0006954, −log10P ≈ 31), response to cytokine (GO:0034097), regulation of inflammatory response (GO:0050727), leukocyte activation (GO:0045321), neutrophil degranulation (R-HSA-6798695), leukocyte migration (GO:0050900), signaling by interleukins (R-HSA-449147), phagocytosis (hsa04145) and the complement system (WP2806) (Figure 1B). Thus, despite arising in different organs and species, both conditions converge on inflammatory and barrier-related biology, with BBBD dominated by a myeloid/innate-immune program.

**Figure 1:**
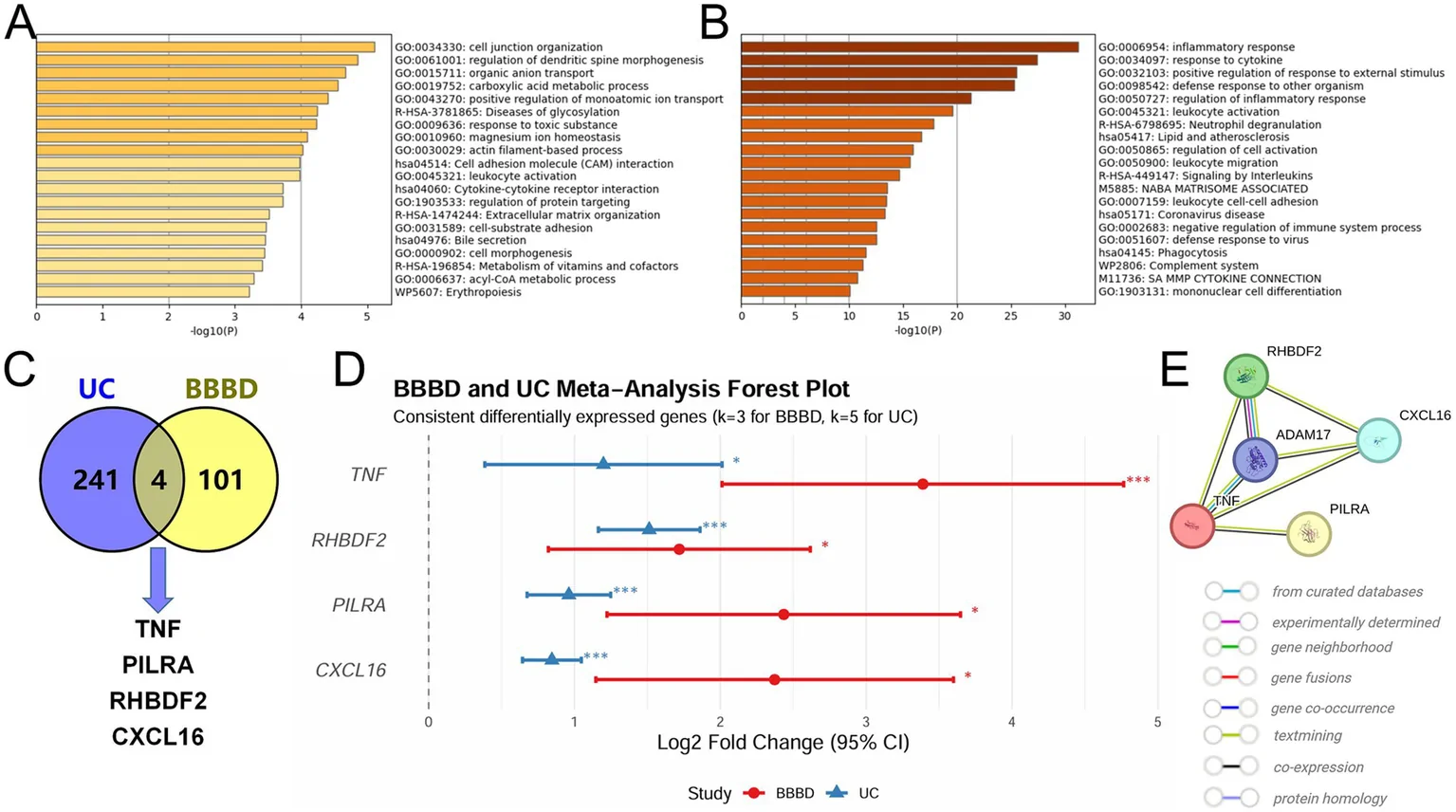
Meta-analysis of ulcerative colitis (UC) and blood–brain barrier disruption (BBBD) transcriptomes identifies four shared up-regulated genes. **(A,B)** Functional enrichment analysis (Metascape) of the robust meta-analytic DEG signatures of UC (**A**, 245 genes from five human cohorts, k = 5) and BBBD (**B**, 141 genes from three mouse cohorts, k = 3). Bars represent the top 20 enriched Gene Ontology (GO), KEGG, Reactome (R-HSA), WikiPathways (WP) and MSigDB (M) terms ranked by −log10(*p* value). **(C)** Venn diagram of the human-mapped BBBD DEG signature (105 human orthologues of 141 mouse DEGs) and the UC DEG signature (245 genes), showing four shared genes: TNF, PILRA, RHBDF2 and CXCL16. **(D)** Forest plot of the random-effects meta-analysis effect sizes (log2 fold change with 95% confidence intervals) of the four shared genes in BBBD (red circles, k = 3) and UC (blue triangles, k = 5). *p < 0.05, **p < 0.01, ***p < 0.001 (meta-analytic *p* values). **(E)** Protein–protein association network (STRING) of the four shared genes with ADAM17 included as a bridging node, given that most of the shared genes are functionally linked to the ADAM17 sheddase pathway (RHBDF2 controls ADAM17 maturation, and TNF and CXCL16 are ADAM17 substrates). Edge colors indicate the evidence channel: known interactions from curated databases (cyan) or experimentally determined (magenta); predicted interactions from gene neighborhood (green), gene fusions (red) or gene co-occurrence (blue); and text mining (yellow-green), co-expression (black) or protein homology (light purple). BBBD, blood–brain barrier disruption; UC, ulcerative colitis; HC, healthy control.

### 3.3 Cross-species integration identifies TNF, RHBDF2, PILRA and CXCL16 as shared up-regulated genes

To bridge the mouse BBBD signature to human UC, the 141 mouse BBBD DEGs were converted to their human orthologues using the HomoloGene database, yielding 105 unique human genes after collapsing many-to-one mappings (the reduction in gene number reflects orthologue merging). Intersection of these human orthologues with the 245 UC DEGs, followed by a three-layer direction-consistency filter (sign consistency within BBBD cohorts, within UC cohorts, and across the two diseases), identified four genes—TNF, RHBDF2, PILRA and CXCL16—that were significantly and concordantly up-regulated in both UC and BBBD (Figure 1C). The random-effects meta-analysis confirmed robust effect sizes for all four genes in both diseases (Figure 1D; Table 2): TNF showed the largest up-regulation in BBBD (β = 3.39, 95% CI 2.01–4.77, FDR = 7.2 × 10^−4^) and was also significantly elevated in UC (β = 1.20, 95% CI 0.39–2.01, FDR = 0.038); RHBDF2 (BBBD β = 1.72, FDR = 0.029; UC β = 1.51, FDR = 2.1 × 10^−15^), PILRA (BBBD β = 2.44, FDR = 0.017; UC β = 0.96, FDR = 2.2 × 10^−9^) and CXCL16 (BBBD β = 2.37, FDR = 0.026; UC β = 0.85, FDR = 1.9 × 10^−14^) were likewise significantly up-regulated in both conditions, with low between-study heterogeneity (I^2^ ≤ 33.3%) for all comparisons except TNF in UC (I^2^ = 64.9%). The between-cohort heterogeneity for TNF was substantial (I^2^ = 64.9%), plausibly reflecting differences in profiling platform, biopsy site relative to inflamed mucosa, disease activity and treatment exposure across cohorts; importantly, the direction of the TNF effect was consistent in all five cohorts.

**Table 2:** Meta-analytic statistics of the four genes shared between UC and BBBD.

Human gene	Mouse gene	BBBD β (log2FC)	BBBD 95% CI	BBBD P	BBBD FDR	BBBD I^2^ (%)	UC β (log2FC)	UC 95% CI	UC P	UC FDR	UC I^2^ (%)
TNF	Tnf	3.389	2.012–4.765	1.4 × 10^−6^	7.2 × 10^−4^	1.5	1.199	0.385–2.012	3.9 × 10^−3^	3.8 × 10^−2^	64.9
RHBDF2	Rhbdf2	1.719	0.821–2.617	1.8 × 10^−4^	2.9 × 10^−2^	5.8	1.513	1.164–1.861	1.9 × 10^−17^	2.1 × 10^−15^	10.5
PILRA	Pilra	2.435	1.223–3.647	8.2 × 10^−5^	1.7 × 10^−2^	0.0	0.962	0.675–1.248	4.8 × 10^−11^	2.2 × 10^−9^	0.0
CXCL16	Cxcl16	2.372	1.146–3.599	1.5 × 10^−4^	2.6 × 10^−2^	33.3	0.845	0.644–1.047	1.9 × 10^−16^	1.9 × 10^−14^	0.0

Because most of the shared genes are mechanistically linked to a common proteolytic pathway—RHBDF2 is the essential chaperone governing the maturation and activity of the sheddase ADAM17, while TNF and CXCL16 are canonical ADAM17 substrates—ADAM17 was incorporated as a bridging node in the protein–protein association network analysis (STRING). The resulting network showed that the four shared genes form a tightly interconnected module organized around ADAM17 (TACE), supported by experimentally determined interactions, curated-database annotations, co-expression and text-mining evidence (Figure 1E). This topology is biologically coherent: RHBDF2 controls ADAM17-dependent shedding of TNF and CXCL16, whereas PILRA acts as an inhibitory immunoreceptor restraining myeloid activation, together suggesting that an RHBDF2–ADAM17–TNF/CXCL16 axis, counterbalanced by PILRA, constitutes a core pathogenic module linking intestinal inflammation to blood–brain barrier disruption.

### 3.4 Single-cell transcriptomic landscape of the UC colonic mucosa

To resolve the cellular context in which the shared UC–BBBD genes operate, we analyzed the public single-cell RNA-sequencing (scRNA-seq) dataset GSE214695, which profiles colonic mucosal cells from six healthy controls (HC) and six UC patients. After quality control and integration, 27,310 cells (13,106 HC and 14,204 UC) were retained and annotated into ten broad lineages—epithelial cells, stromal cells, endothelial cells, B cells, plasma cells, CD4 T cells, CD8 T cells, other T cells, NK cells & ILCs, and myeloid cells—visualized by UMAP (Figure 2A). Cell-type composition differed markedly between groups at both the per-sample and the pooled level (Figures 2B,C): the UC mucosa was characterized by a profound loss of epithelial cells (41.1% of all cells in HC vs. 8.4% in UC) and a compensatory expansion of immune lineages, most prominently plasma cells (22.3% vs. 44.7%), myeloid cells (3.7% vs. 7.8%), B cells (2.4% vs. 6.1%) and CD4 T cells (8.9% vs. 14.1%), recapitulating the epithelial damage and immune infiltration that define active UC.

**Figure 2:**
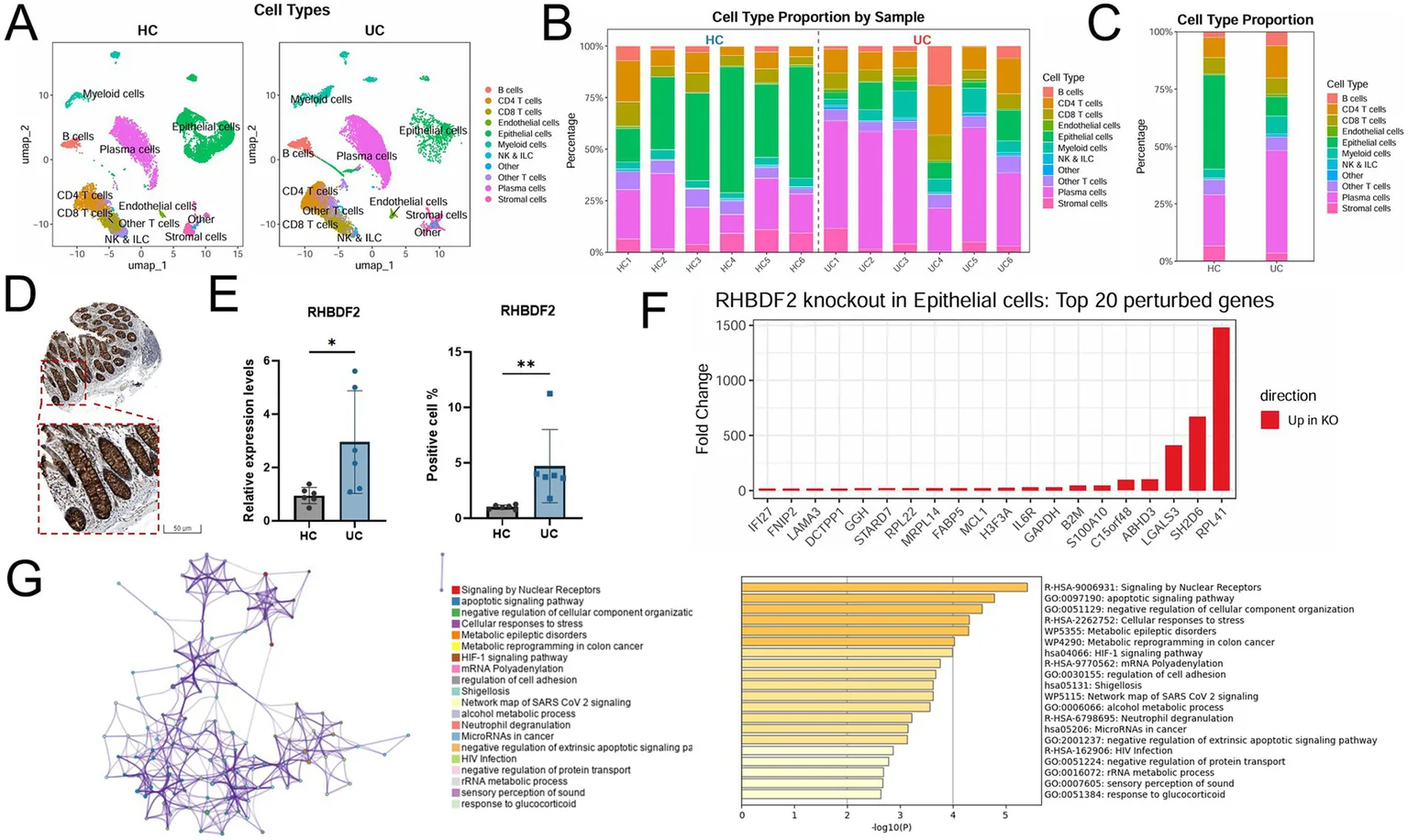
Single-cell analysis identifies epithelial RHBDF2 as a dysregulated gatekeeper in UC. **(A)** UMAP visualization of integrated scRNA-seq profiles of colonic mucosal cells from six healthy controls (HC) and six UC patients (GSE214695), colored by ten broad cell-type annotations. **(B,C)** Stacked bar plots of cell-type proportions in individual samples **(B)** and in pooled HC versus UC groups **(C)**. **(D)** Representative RHBDF2 immunohistochemistry staining of normal human colon from the Human Protein Atlas, showing predominant staining of glandular epithelial cells (scale bar, 50 μm; lower panel, magnified view of the boxed region). **(E)** RHBDF2 expression level (left) and percentage of RHBDF2-positive cells (right) in epithelial cells of HC versus UC samples (*n* = 6 per group). Bars represent the mean ± SD, and dots represent individual samples; statistical significance was assessed by unpaired two-tailed Student’s t-test. *p < 0.05, **p < 0.01. **(F)** Top 20 genes perturbed by in-silico knockout of RHBDF2 in epithelial cells (scTenifoldKnk); bars show the fold change (FC) of each gene upon knockout, and all significantly perturbed genes were up-regulated in the knockout (red). **(G)** Metascape enrichment network (left) and top 20 enriched terms [right, ranked by −log10(*p* value)] of the 67 genes significantly perturbed by RHBDF2 virtual knockout (*p* < 0.05). HC, healthy control; UC, ulcerative colitis; HPA, Human Protein Atlas.

### 3.5 RHBDF2 is predominantly expressed in colonic epithelial cells and is up-regulated in UC

Among the four shared UC–BBBD genes, we prioritized RHBDF2 for single-cell validation because of its established role as the essential activator of ADAM17-mediated TNF/CXCL16 shedding. To the best of our knowledge, no prior study has reported an association between RHBDF2 and BBBD. We therefore selected RHBDF2 for further validation, a choice that lends novelty to our study. Consistent with a predominantly epithelial site of action, immunohistochemistry data from the Human Protein Atlas (HPA) showed strong RHBDF2 protein staining in the glandular epithelial cells of the colonic mucosa (Figure 2D). In GSE214695, RHBDF2 was significantly up-regulated in UC epithelial cells compared with HC (mean normalized expression 0.037 vs. 0.017, log2FC = 1.10, adjusted *p* = 2.65 × 10^−22^, two-sided Wilcoxon rank-sum test), and the fraction of RHBDF2-positive epithelial cells increased from 1.4% in HC to 6.1% in UC. At the sample level (*n* = 6 per group), both the mean RHBDF2 expression and the percentage of RHBDF2-positive epithelial cells were significantly higher in UC than in HC (Figure 2E; unpaired two-tailed Student’s *t*-test, *p < 0.05 and **p < 0.01, respectively). A cell-type–wide survey (Table 3) confirmed that the epithelial compartment showed the most robust UC-associated induction of RHBDF2, whereas RHBDF2 was modestly down-regulated in plasma cells and CD4 T cells and unchanged in the remaining lineages.

**Table 3:** Cell-type–resolved comparison of RHBDF2 expression between HC and UC in GSE214695.

Cell type	HC mean	UC mean	log2FC (UC/HC)	*p* value	Adjusted *p*	HC cells (*n*)	UC cells (*n*)	Significance
Epithelial cells	0.017	0.037	1.10	2.4 × 10^−23^	2.7 × 10^−22^	5,382	1,193	***
Plasma cells	0.067	0.039	−0.76	3.7 × 10^−15^	2.1 × 10^−14^	2,924	6,351	***
CD4 T cells	0.085	0.046	−0.88	5.8 × 10^−5^	2.1 × 10^−4^	1,161	1,999	***
NK & ILC	0.079	0.254	1.68	3.5 × 10^−2^	0.096	68	98	ns
Other	0.013	0.087	2.67	0.094	0.173	43	104	ns
Other T cells	0.081	0.117	0.53	0.087	0.173	861	829	ns
Endothelial cells	0.041	0.109	1.40	0.163	0.256	70	159	ns
Stromal cells	0.027	0.047	0.80	0.194	0.267	870	501	ns
Myeloid cells	0.135	0.167	0.31	0.230	0.281	486	1,102	ns
CD8 T cells	0.080	0.095	0.24	0.698	0.768	920	1,005	ns
B cells	0.211	0.204	−0.04	0.794	0.794	321	863	ns

*p* values were calculated by two-sided Wilcoxon rank-sum test at the single-cell level and adjusted for multiple testing using the Benjamini–Hochberg method; ***Adjusted *p* < 0.001; ns, not significant. Mean expression values are normalized expression levels averaged over all cells of the indicated type.

### 3.6 Virtual knockout of RHBDF2 in epithelial cells de-represses stress, apoptotic and inflammatory programs

To infer the causal regulatory function of epithelial RHBDF2, we performed an in-silico gene knockout using scTenifoldKnk on the epithelial-cell regulatory network. Virtual deletion of RHBDF2 significantly perturbed 23 genes at FDR < 0.05 (67 genes at *p* < 0.05), all of which were up-regulated upon knockout (Figure 2F; Table 4). The fold change reported by scTenifoldKnk is a network-distance-based metric of regulatory rewiring rather than an expression fold change (Table 4), and the significantly perturbed genes spanned a wide range of baseline expression levels in epithelial cells (Table 4). The most strongly de-repressed genes included RPL41 (FC ≈ 1,478, FDR = 5.9 × 10^−320^), SH2D6 (FC ≈ 670), LGALS3 (FC ≈ 410), ABHD3, C15orf48, S100A10, B2M, GAPDH, IL6R, H3F3A and MCL1, suggesting that RHBDF2 is predicted to restrain a broad transcriptional program in epithelial cells, a hypothesis that awaits experimental perturbation. Metascape enrichment analysis of the 67 significantly perturbed genes (*p* < 0.05) (Figure 2G) revealed over-representation of signaling by nuclear receptors (R-HSA-9006931), apoptotic signaling pathway (GO:0097190), cellular responses to stress (R-HSA-2262752), HIF-1 signaling (hsa04066), regulation of cell adhesion (GO:0030155), neutrophil degranulation (R-HSA-6798695) and negative regulation of extrinsic apoptotic signaling (GO:2001237). These findings suggest that epithelial RHBDF2 functions as a gatekeeper of mucosal homeostasis, and that its dysregulation in UC couples epithelial stress/apoptosis to innate immune activation—a mechanism consistent with its proposed role at the gut–brain barrier interface.

**Table 4:** Genes significantly perturbed by scTenifoldKnk virtual knockout of RHBDF2 in epithelial cells (FDR < 0.05; all up-regulated upon knockout), with baseline expression in epithelial cells of GSE214695.

Gene	Z score	Fold change	*p* value	FDR	Mean expression (epithelial)	Cells expressing (%)
RPL41	4.49	1478.5	2.0 × 10^−323^	5.9 × 10^−320^	30.17	87.44
SH2D6	4.05	670.2	8.9 × 10^−148^	1.3 × 10^−144^	3.12	5.81
LGALS3	3.79	409.8	4.1 × 10^−91^	4.1 × 10^−88^	17.05	72.23
ABHD3	3.09	101.2	8.2 × 10^−24^	6.1 × 10^−21^	0.53	21.02
C15orf48	3.06	95.2	1.7 × 10^−22^	1.0 × 10^−19^	7.96	63.42
S100A10	2.72	46.2	1.1 × 10^−11^	5.3 × 10^−9^	11.77	69.37
B2M	2.71	45.1	1.8 × 10^−11^	7.9 × 10^−9^	41.9	88.37
GAPDH	2.51	29.3	6.2 × 10^−8^	2.3 × 10^−5^	11.9	76.46
IL6R	2.5	28.6	8.8 × 10^−8^	2.9 × 10^−5^	0.18	9.64
H3F3A	2.47	26.5	2.6 × 10^−7^	7.7 × 10^−5^	7.75	69.64
MCL1	2.39	22.4	2.2 × 10^−6^	6.0 × 10^−4^	1.75	38.97
FABP5	2.35	20.6	5.7 × 10^−6^	1.4 × 10^−3^	1.44	27.89
MRPL14	2.34	20.2	6.9 × 10^−6^	1.6 × 10^−3^	0.61	25.67
RPL22	2.34	19.8	8.4 × 10^−6^	1.8 × 10^−3^	5.64	52.9
STARD7	2.32	19.3	1.1 × 10^−5^	2.3 × 10^−3^	0.19	10.08
GGH	2.31	18.9	1.4 × 10^−5^	2.7 × 10^−3^	0.16	9.86
DCTPP1	2.3	18.4	1.8 × 10^−5^	3.1 × 10^−3^	0.5	19.32
LAMA3	2.28	17.6	2.7 × 10^−5^	4.5 × 10^−3^	2.03	26.11
FNIP2	2.28	17.5	2.9 × 10^−5^	4.6 × 10^−3^	0.29	13.34
IFI27	2.26	16.5	4.7 × 10^−5^	7.1 × 10^−3^	18.56	70.78
COX7A2	2.18	13.9	2.0 × 10^−4^	2.8 × 10^−2^	3.78	54.08
RPS6	2.18	13.7	2.1 × 10^−4^	2.9 × 10^−2^	10.29	64.46
GUCA2A	2.16	13.3	2.6 × 10^−4^	3.4 × 10^−2^	15.47	35.92

In scTenifoldKnk, the fold change quantifies the change in a gene’s position within the gene-regulatory network after virtual knockout (a network-distance-based metric), not the change in expression level; its magnitude can be mathematically inflated for genes with small pre-knockout network distances and should not be interpreted as an expression fold change. Extreme fold changes occur in both highly expressed genes (e.g., RPL41, expressed in 87.4% of epithelial cells) and lowly expressed genes (e.g., SH2D6, 5.8%), confirming that they reflect the distance metric rather than baseline expression. Mean expression (log-normalized data, linear scale) and the percentage of expressing cells were computed from epithelial cells of GSE214695 (6 HC + 6 UC samples). Interpretation should rely primarily on Z scores and FDR.

### 3.7 SMR analysis links genetically predicted RHBDF2 and PILRA expression to dementia risk

Because BBBD is an early and prominent feature of dementia pathogenesis, we next asked whether the shared UC–BBBD genes have causal relevance to dementia. We performed summary-data-based Mendelian randomization (SMR) integrating whole-blood cis-eQTL summary statistics from eQTLGen with FinnGen GWAS summary statistics for four dementia endpoints (all-cause dementia, Alzheimer’s dementia, Parkinson’s dementia and vascular dementia), testing the four shared genes (TNF, RHBDF2, PILRA and CXCL16) with the top cis-eQTL of each gene as the instrumental variable (Figure 3A; Table 5). After Benjamini–Hochberg correction across the four genes within each phenotype, genetically predicted higher expression of PILRA and RHBDF2 was significantly associated with increased risk of all-cause dementia (PILRA: b_SMR = 0.43, 95% CI 0.17–0.70, P_SMR = 1.3 × 10^−3^, P_BH = 0.0053; RHBDF2: b_SMR = 0.27, 95% CI 0.04–0.49, P_SMR = 0.0185, P_BH = 0.037), and both genes were also significantly associated with Alzheimer’s dementia (RHBDF2: b_SMR = 0.59, 95% CI 0.21–0.97, P_SMR = 2.2 × 10^−3^, P_BH = 0.0086; PILRA: b_SMR = 0.59, 95% CI 0.15–1.03, P_SMR = 8.0 × 10^−3^, P_BH = 0.016). In contrast, no significant association was detected for Parkinson’s dementia or vascular dementia, and neither TNF nor CXCL16 was associated with any dementia endpoint after correction. The HEIDI test, which distinguishes pleiotropy from linkage, supported a shared causal variant for the PILRA–Alzheimer’s dementia association (P_HEIDI = 0.094), whereas the remaining significant associations showed evidence of heterogeneity (P_HEIDI < 0.05) and should thus be interpreted with appropriate caution. SMR effect-size plots at the RHBDF2 locus illustrated the concordance between cis-eQTL and GWAS effect sizes around the top cis-eQTL rs4238992 across the four dementia endpoints (Figure 3B). Together, these genetic data support a causal, risk-increasing role of elevated RHBDF2 and PILRA expression in all-cause and Alzheimer’s dementia, strengthening the hypothesis that the UC–BBBD shared genes also contribute to downstream neurodegenerative sequelae of chronic barrier dysfunction.

**Figure 3:**
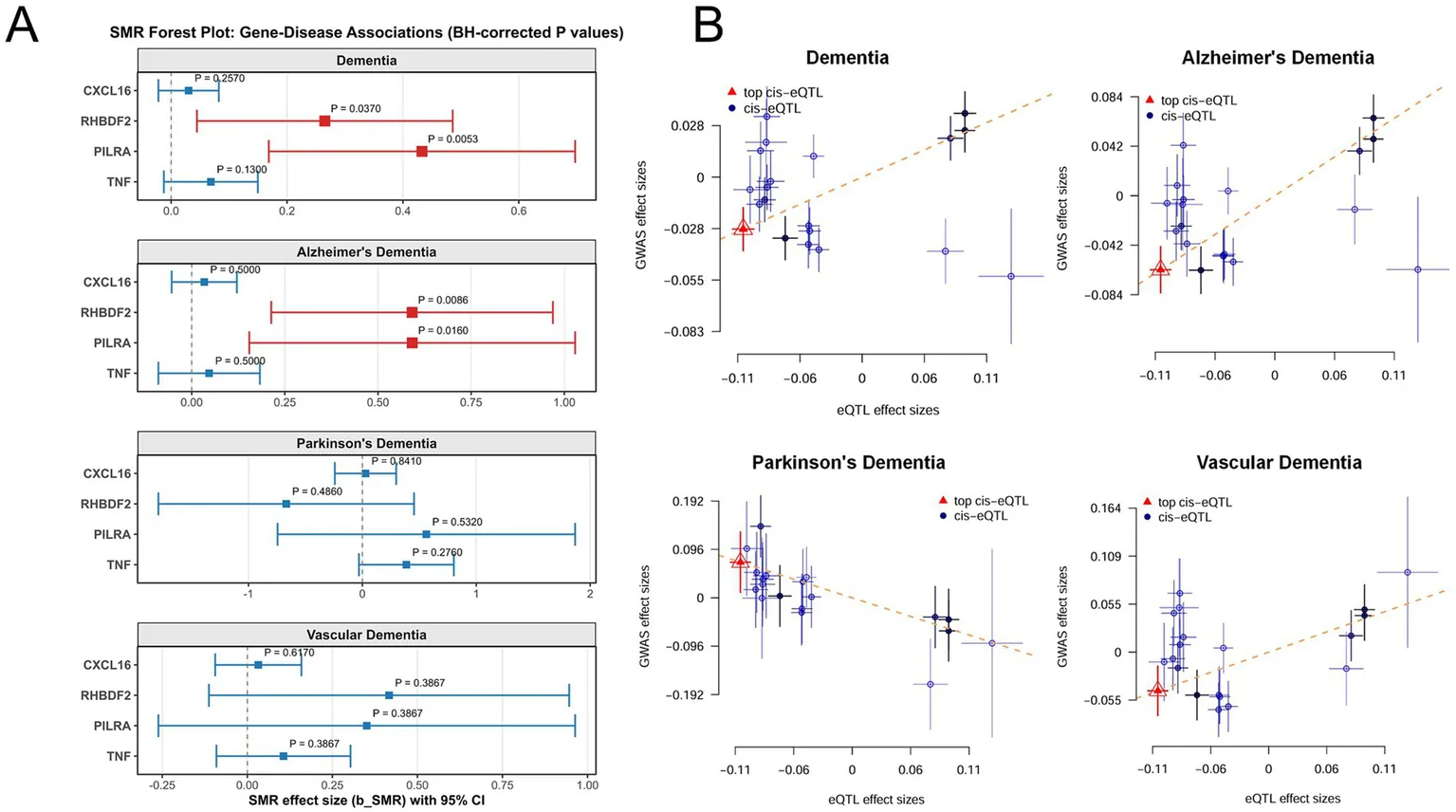
Summary-data-based Mendelian randomization (SMR) links RHBDF2 and PILRA expression to dementia risk. **(A)** Forest plot of SMR effect sizes (b_SMR with 95% confidence intervals) of the four shared UC–BBBD genes (TNF, RHBDF2, PILRA, CXCL16) across four FinnGen dementia endpoints (all-cause dementia, Alzheimer’s dementia, Parkinson’s dementia and vascular dementia), using whole-blood cis-eQTL data from eQTLGen and the top cis-eQTL of each gene as the instrumental variable. *p* values are Benjamini–Hochberg-corrected across the four genes within each phenotype; red, significant (P_BH < 0.05); blue, not significant. **(B)** SMR effect-size plots for RHBDF2 at each dementia endpoint, showing the correlation between cis-eQTL effect sizes (x-axis) and GWAS effect sizes (y-axis) of SNPs surrounding the RHBDF2 locus. The top cis-eQTL (rs4238992) is shown as a red triangle; filled navy circles denote cis-eQTLs passing the SMR threshold. Plots were generated with the SMR plotting tool (Zhu et al., 2016). SMR, summary-data-based Mendelian randomization; eQTL, expression quantitative trait locus; GWAS, genome-wide association study.

**Table 5:** SMR analysis of the four shared UC–BBBD genes across four FinnGen dementia endpoints (eQTLGen whole-blood cis-eQTL).

Phenotype	Gene	Top cis-eQTL	b_SMR	SE	95% CI	P_SMR	P_BH	P_HEIDI	*n* SNPs (HEIDI)	Significant (BH < 0.05)
Dementia	TNF	rs1121800	0.069	0.041	−0.013 to 0.150	0.098	0.130	0.032	20	No
Dementia	PILRA	rs2005763	0.433	0.135	0.169 to 0.697	1.3 × 10^−3^	0.0053	0.047	20	Yes
Dementia	RHBDF2	rs4238992	0.265	0.113	0.045 to 0.486	0.019	0.037	0.0035	20	Yes
Dementia	CXCL16	rs1050998	0.030	0.027	−0.022 to 0.082	0.257	0.257	0.095	20	No
Alzheimer’s dementia	TNF	rs1121800	0.047	0.069	−0.089 to 0.183	0.500	0.500	0.035	20	No
Alzheimer’s dementia	PILRA	rs2005763	0.592	0.223	0.155 to 1.029	8.0 × 10^−3^	0.016	0.094	20	Yes
Alzheimer’s dementia	RHBDF2	rs4238992	0.592	0.193	0.214 to 0.969	2.2 × 10^−3^	0.0086	0.0090	20	Yes
Alzheimer’s dementia	CXCL16	rs1050998	0.034	0.045	−0.054 to 0.121	0.451	0.500	0.115	20	No
Parkinson’s dementia	TNF	rs1121800	0.386	0.213	−0.030 to 0.803	0.069	0.276	0.873	20	No
Parkinson’s dementia	PILRA	rs2005763	0.562	0.666	−0.745 to 1.868	0.399	0.532	0.841	20	No
Parkinson’s dementia	RHBDF2	rs4238992	−0.668	0.573	−1.791 to 0.454	0.243	0.486	0.701	20	No
Parkinson’s dementia	CXCL16	rs1050998	0.027	0.137	−0.242 to 0.297	0.841	0.841	0.572	20	No
Vascular dementia	TNF	rs1121800	0.106	0.101	−0.091 to 0.303	0.290	0.387	0.357	20	No
Vascular dementia	PILRA	rs2005763	0.351	0.312	−0.261 to 0.964	0.261	0.387	0.797	20	No
Vascular dementia	RHBDF2	rs4238992	0.417	0.270	−0.113 to 0.946	0.123	0.387	0.069	20	No
Vascular dementia	CXCL16	rs1050998	0.032	0.065	−0.094 to 0.159	0.617	0.617	0.573	20	No

b_SMR, SMR effect size (positive values indicate that genetically predicted higher gene expression is associated with higher disease risk); P_BH, Benjamini–Hochberg-adjusted *p* value across the four genes within each phenotype; P_HEIDI, *p* value of the HEIDI test (P_HEIDI ≥ 0.05 indicates no significant heterogeneity, consistent with pleiotropy at a shared causal variant).

### 3.8 Endothelial RHBDF2 exerts the strongest regulatory impact on the blood–brain barrier

Finally, to test whether the gut-relevant gene RHBDF2 also plays a regulatory role directly at the human blood–brain barrier (BBB), we analyzed GSE208074, a paired single-cell RNA-seq dataset of peritumoral brain biopsies from six recurrent glioblastoma patients undergoing low-intensity pulsed ultrasound (LIPU)-mediated BBB opening, in which a sonicated (BBB-opened) sample and a non-sonicated (BBB-intact) sample were obtained from each patient. After integration, 86,403 cells were annotated into eleven major brain cell types, including the three core cellular constituents of the neurovascular unit—endothelial cells, pericytes and astrocytes (Supplementary Figure S1A). RHBDF2 was broadly expressed across neural and immune compartments, with the highest positive-cell rates in microglia/macrophages and astrocytes, and detectable expression in endothelial cells and pericytes, at broadly comparable levels between BBB-opened and BBB-intact samples (Supplementary Figure S1B).

We then performed scTenifoldKnk in-silico knockout of RHBDF2 separately in the three neurovascular-unit cell types. Virtual knockout in endothelial cells perturbed by far the largest transcriptional program—78 genes (*p* < 0.05)—compared with only 14 genes in pericytes and 2 genes in astrocytes (Figure 4A; Table 6), suggesting that endothelial RHBDF2 may carry the dominant regulatory weight at the BBB. The endothelial RHBDF2-regulated genes formed a densely interconnected functional module in GeneMANIA analysis (Figure 4B), which included the shared UC–BBBD gene CXCL16, the RHBDF2 effector protease ADAM17, complement components (C1QA, C1QB, C1QC, C3), microglial/myeloid regulators (TYROBP, TREM2, AIF1, FCER1G, LAPTM5) and vascular/endothelial factors (CAV1, CAV2, CAVIN1, S1PR1, APOLD1, TM4SF1). Metascape enrichment analysis of the 78 genes (Figure 4C) highlighted neutrophil degranulation (R-HSA-6798695), regulation of cell activation (GO:0050865), positive regulation of cytokine production (GO:0001819), the microglia pathogen phagocytosis pathway (WP3937), the TYROBP causal network in microglia (WP3945), VEGFA–VEGFR2 signaling (WP3888), cell surface interactions at the vascular wall (R-HSA-202733), complement-mediated synapse pruning (GO:0150062) and regulation of microglial cell activation (GO:1903978). These computational predictions position endothelial RHBDF2 as a candidate regulatory hub of the neurovascular unit that restrains innate-immune, complement and angiogenic programs at the human BBB, providing a mechanistic bridge between its up-regulation in the UC epithelium and the blood–brain barrier dysfunction observed in BBBD models.

**Figure 4:**
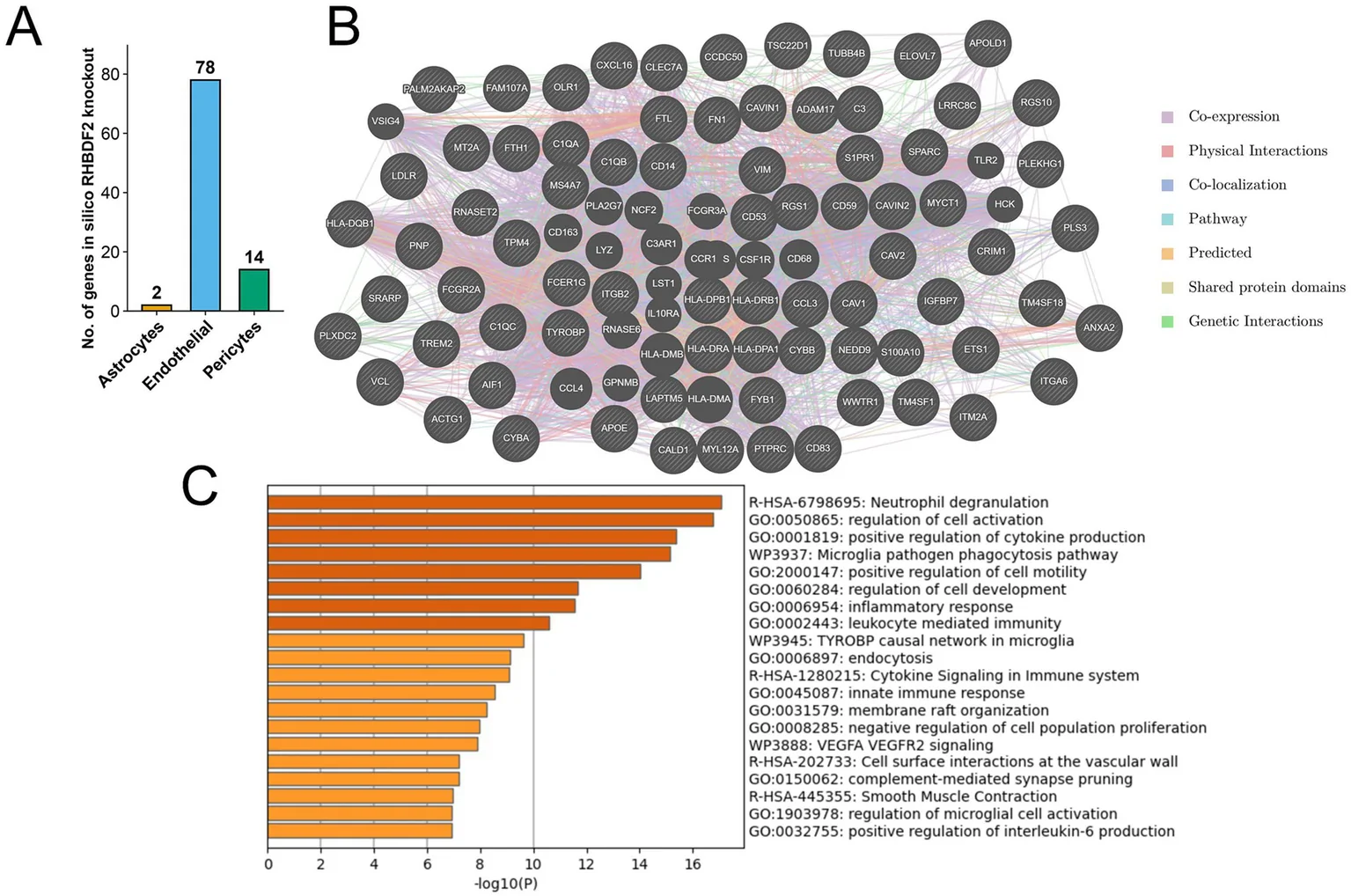
In-silico knockout of RHBDF2 in the human blood–brain barrier identifies endothelial cells as the dominant regulatory target. **(A)** Number of genes significantly perturbed (*p* < 0.05) by scTenifoldKnk virtual knockout of RHBDF2 in astrocytes (*n* = 2), endothelial cells (*n* = 78) and pericytes (*n* = 14) from the paired human BBB dataset GSE208074 (peritumoral brain biopsies of six recurrent glioblastoma patients, LIPU-mediated BBB opening). **(B)** GeneMANIA interaction network of the 78 genes perturbed by endothelial RHBDF2 knockout. Edge colors indicate the evidence type: co-expression, physical interactions, co-localization, pathway, predicted interactions, shared protein domains and genetic interactions. **(C)** Metascape enrichment analysis of the 78 endothelial RHBDF2-regulated genes; bars show the top 20 enriched terms ranked by −log10 (*p* value). LIPU, low-intensity pulsed ultrasound; BBB, blood–brain barrier.

**Table 6:** The 78 genes significantly perturbed (*p* < 0.05) by scTenifoldKnk virtual knockout of RHBDF2 in endothelial cells of GSE208074 (ranked by fold change).

Gene	Z score	Fold change	*p* value	Adjusted *p*
MYL12A	2.75	1746.1	<1 × 10^−300^	<1 × 10^−300^
CD59	1.92	21.0	4.6 × 10^−6^	4.1 × 10^−3^
VIM	1.88	16.1	6.1 × 10^−5^	3.4 × 10^−2^
TM4SF1	1.88	16.0	6.2 × 10^−5^	3.4 × 10^−2^
TYROBP	1.84	12.8	3.4 × 10^−4^	1.6 × 10^−1^
APOLD1	1.83	12.3	4.6 × 10^−4^	1.7 × 10^−1^
MT2A	1.83	12.1	5.0 × 10^−4^	1.7 × 10^−1^
S100A10	1.81	10.8	9.9 × 10^−4^	2.5 × 10^−1^
WWTR1	1.81	10.8	1.0 × 10^−3^	2.5 × 10^−1^
C1QB	1.81	10.7	1.1 × 10^−3^	2.5 × 10^−1^
CAVIN1	1.81	10.6	1.1 × 10^−3^	2.5 × 10^−1^
CD74	1.81	10.5	1.2 × 10^−3^	2.5 × 10^−1^
S1PR1	1.80	10.2	1.4 × 10^−3^	2.7 × 10^−1^
MYCT1	1.79	9.6	1.9 × 10^−3^	3.5 × 10^−1^
ITM2A	1.79	9.3	2.3 × 10^−3^	3.8 × 10^−1^
ANXA2	1.78	9.3	2.3 × 10^−3^	3.8 × 10^−1^
CAV2	1.78	8.9	2.8 × 10^−3^	4.3 × 10^−1^
C1QC	1.78	8.8	3.0 × 10^−3^	4.3 × 10^−1^
TSC22D1	1.77	8.7	3.3 × 10^−3^	4.5 × 10^−1^
C1QA	1.77	8.3	4.0 × 10^−3^	4.9 × 10^−1^
AIF1	1.77	8.2	4.1 × 10^−3^	4.9 × 10^−1^
FAM107A	1.77	8.2	4.1 × 10^−3^	4.9 × 10^−1^
LAPTM5	1.76	8.1	4.4 × 10^−3^	5.0 × 10^−1^
CAV1	1.76	8.0	4.7 × 10^−3^	5.1 × 10^−1^
RNASET2	1.76	7.9	5.1 × 10^−3^	5.3 × 10^−1^
CD83	1.75	7.7	5.5 × 10^−3^	5.5 × 10^−1^
MS4A7	1.75	7.5	6.2 × 10^−3^	6.1 × 10^−1^
PLXDC2	1.75	7.4	6.5 × 10^−3^	6.2 × 10^−1^
FN1	1.74	7.2	7.1 × 10^−3^	6.5 × 10^−1^
PALM2AKAP2	1.74	7.1	7.5 × 10^−3^	6.6 × 10^−1^
NEDD9	1.74	7.0	8.3 × 10^−3^	6.6 × 10^−1^
HLA-DRA	1.74	7.0	8.3 × 10^−3^	6.6 × 10^−1^
TM4SF18	1.74	7.0	8.3 × 10^−3^	6.6 × 10^−1^
FCER1G	1.74	6.9	8.6 × 10^−3^	6.6 × 10^−1^
SPARC	1.73	6.9	8.8 × 10^−3^	6.6 × 10^−1^
OLR1	1.73	6.8	9.0 × 10^−3^	6.6 × 10^−1^
ETS1	1.73	6.5	1.1 × 10^−2^	7.4 × 10^−1^
IGFBP7	1.73	6.5	1.1 × 10^−2^	7.4 × 10^−1^
CALD1	1.73	6.5	1.1 × 10^−2^	7.4 × 10^−1^
CCL3	1.72	6.5	1.1 × 10^−2^	7.4 × 10^−1^
LRRC8C	1.72	6.2	1.3 × 10^−2^	8.2 × 10^−1^
TPM4	1.72	6.2	1.3 × 10^−2^	8.3 × 10^−1^
PLS3	1.71	6.0	1.4 × 10^−2^	8.7 × 10^−1^
SRARP	1.71	5.9	1.5 × 10^−2^	9.1 × 10^−1^
ACTG1	1.71	5.7	1.7 × 10^−2^	9.6 × 10^−1^
FTH1	1.71	5.7	1.7 × 10^−2^	9.6 × 10^−1^
CAVIN2	1.70	5.7	1.7 × 10^−2^	9.6 × 10^−1^
CYBB	1.70	5.6	1.8 × 10^−2^	9.6 × 10^−1^
CYBA	1.70	5.6	1.8 × 10^−2^	9.6 × 10^−1^
CLEC7A	1.70	5.6	1.8 × 10^−2^	9.6 × 10^−1^
TREM2	1.70	5.5	1.9 × 10^−2^	9.6 × 10^−1^
PTPRC	1.70	5.5	1.9 × 10^−2^	9.6 × 10^−1^
RGS1	1.70	5.5	1.9 × 10^−2^	9.6 × 10^−1^
APOE	1.70	5.4	2.0 × 10^−2^	9.8 × 10^−1^
ITGA6	1.70	5.4	2.0 × 10^−2^	9.8 × 10^−1^
PSAP	1.70	5.4	2.0 × 10^−2^	9.8 × 10^−1^
C3	1.69	5.2	2.2 × 10^−2^	1.0 × 10^0^
FCGR2A	1.69	5.1	2.4 × 10^−2^	1.0 × 10^0^
GPX1	1.69	5.1	2.4 × 10^−2^	1.0 × 10^0^
ELOVL7	1.69	5.1	2.4 × 10^−2^	1.0 × 10^0^
HLA-DPA1	1.68	5.0	2.5 × 10^−2^	1.0 × 10^0^
RGS10	1.68	5.0	2.6 × 10^−2^	1.0 × 10^0^
CCDC50	1.68	4.9	2.6 × 10^−2^	1.0 × 10^0^
ITGB2	1.68	4.9	2.6 × 10^−2^	1.0 × 10^0^
CRIM1	1.68	4.8	2.9 × 10^−2^	1.0 × 10^0^
FTL	1.68	4.8	2.9 × 10^−2^	1.0 × 10^0^
CD14	1.67	4.7	3.0 × 10^−2^	1.0 × 10^0^
TUBB4B	1.67	4.7	3.1 × 10^−2^	1.0 × 10^0^
PNP	1.67	4.6	3.1 × 10^−2^	1.0 × 10^0^
FYB1	1.67	4.6	3.1 × 10^−2^	1.0 × 10^0^
HLA-DPB1	1.67	4.5	3.4 × 10^−2^	1.0 × 10^0^
HLA-DRB1	1.66	4.4	3.5 × 10^−2^	1.0 × 10^0^
CD53	1.66	4.4	3.6 × 10^−2^	1.0 × 10^0^
PLEKHG1	1.66	4.4	3.6 × 10^−2^	1.0 × 10^0^
HLA-DQB1	1.66	4.2	4.1 × 10^−2^	1.0 × 10^0^
VCL	1.66	4.2	4.1 × 10^−2^	1.0 × 10^0^
LDLR	1.65	4.0	4.5 × 10^−2^	1.0 × 10^0^
CXCL16	1.64	3.9	4.9 × 10^−2^	1.0 × 10^0^

Adjusted *p* values were calculated by the Benjamini–Hochberg method; four genes (MYL12A, CD59, VIM, TM4SF1) remained significant at FDR < 0.05.

### 3.9 Preventive pharmacological inhibition of ADAM17 attenuates DSS-induced colitis and blood–brain barrier disruption in mice

RHBDF2 (iRhom2) is best characterized as the essential activating chaperone of ADAM17, being required for ADAM17 maturation and trafficking and thereby controlling stimulated TNFα release in immune and epithelial cells (Issuree et al., 2013; Maretzky et al., 2013; Giese et al., 2021); importantly, iRhom2 is up-regulated in the inflamed colonic mucosa of patients with IBD, and its deficiency ameliorates experimental colitis (Kim et al., 2020). This well-defined RHBDF2–ADAM17 functional hierarchy offered a pharmacologically tractable entry point for in-vivo testing: beyond verifying RHBDF2 expression in UC-associated blood–brain barrier disruption, we asked whether blockade of its principal downstream effector, ADAM17, would attenuate UC-mediated BBB injury. To experimentally validate that RHBDF2 drives UC-associated BBB injury through the ADAM17–TNFα pathway, we established a dextran sulfate sodium (DSS)-induced colitis mouse model with pharmacological blockade of ADAM17. Mice were allocated to three groups: a normal control group (NC) receiving regular drinking water, a model group (Model) receiving 2.5% DSS in drinking water for 7 consecutive days, and a treatment group (Treat) receiving the same 2.5% DSS regimen together with the ADAM17 inhibitor GW280264X (500 μg/kg, administered intraperitoneally once daily from the first day of DSS exposure, i.e., a preventive regimen) (Huner Yigit et al., 2025; Yu et al., 2025). All mice received a standardized tail-vein injection of Evans blue before tissue harvesting to assess blood–brain barrier permeability.

DSS administration induced a robust colitis phenotype, confirming successful modeling (Figures 5A–C). Whereas NC mice steadily gained body weight over the 7-day period, Model mice progressively lost weight from day 3 onwards, with the difference becoming significant at the later time points (*p* < 0.01–0.001 versus NC); GW280264X treatment largely prevented weight loss, and Treat mice maintained body weight close to baseline throughout the experiment (Figure 5A). The disease activity index (DAI) remained at zero in NC mice, rose progressively in Model mice (*p* < 0.001–0.0001 versus NC from day 4 onwards), and was markedly attenuated in Treat mice, in which the DAI peaked transiently around day 5 and declined thereafter (Figure 5B). Consistent with the clinical course, colonic mRNA levels of the pro-inflammatory cytokines Tnf, Il6 and Il1b were strongly induced in Model mice and were significantly attenuated by GW280264X treatment (Figure 5C).

**Figure 5:**
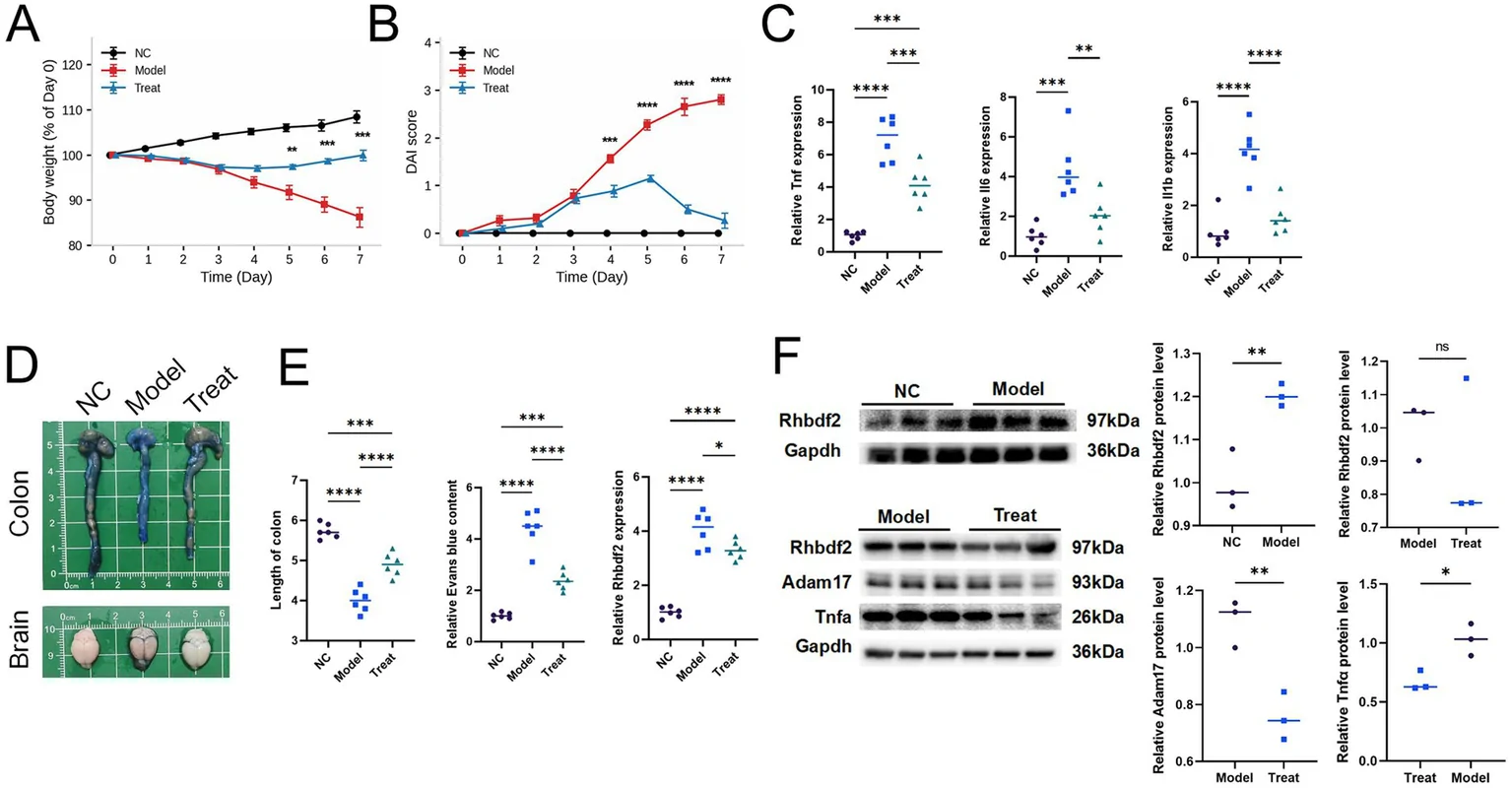
Pharmacological inhibition of ADAM17 with GW280264X attenuates DSS-induced colitis and blood–brain barrier disruption in mice. Mice were divided into three groups: normal control (NC, regular drinking water), model (Model, 2.5% DSS in drinking water for 7 days) and treatment (Treat, 2.5% DSS for 7 days plus once-daily intraperitoneal administration of the ADAM17 inhibitor GW280264X at 500 μg/kg starting on the first day of DSS exposure, i.e., a preventive regimen). All mice received a tail-vein injection of Evans blue before tissue harvesting. **(A)** Body weight changes (expressed as percentage of day-0 body weight) over the 7-day experiment. **(B)** Disease activity index (DAI) scores over the 7-day experiment. **(C)** RT-qPCR analysis of Tnf, Il6 and Il1b mRNA expression in colon tissue. **(D)** Representative gross morphology of Evans blue–perfused colons (top) and brains (bottom) from the three groups. **(E)** Quantification of colon length (left), relative brain Evans blue content determined by 620-nm spectrophotometry (middle), and colonic Rhbdf2 mRNA expression by RT-qPCR (right). **(F)** Western blot analysis of brain tissue, showing elevated Rhbdf2 protein in Model versus NC mice (top), and reduced Rhbdf2, Adam17 and Tnfα protein levels after GW280264X treatment (bottom); Gapdh served as the loading control. Molecular weights are indicated on the right. Data are presented as mean ± SEM **(A,B)** or mean with individual data points **(C,E)**. Statistical analysis was performed by two-way repeated-measures ANOVA followed by Tukey’s multiple comparisons test **(A,B)** or one-way ANOVA followed by Tukey’s multiple comparisons test **(C,E)**. *p < 0.05, **p < 0.01, ***p < 0.001, ****p < 0.0001. DSS, dextran sulfate sodium; DAI, disease activity index; RT-qPCR, reverse-transcription quantitative PCR.

Gross examination revealed marked colonic shortening and pronounced Evans blue extravasation in Model mice, together with visible blue discoloration of the brain parenchyma indicating BBB leakage, both of which were alleviated in the Treat group (Figure 5D). Quantitative analysis confirmed significant colon shortening in Model versus NC mice (*p* < 0.0001), which was partially rescued by GW280264X (*p* < 0.001 versus Model) (Figure 5E, left). Spectrophotometric quantification of Evans blue at 620 nm demonstrated a marked increase in brain dye content in Model mice (*p* < 0.0001 versus NC)—directly evidencing BBB disruption in DSS colitis—which was significantly reduced by ADAM17 inhibition (*p* < 0.001 versus Model) (Figure 5E, middle). Notably, Rhbdf2 mRNA was significantly up-regulated in the colon of DSS-treated mice (*p* < 0.0001 versus NC) and was partially suppressed by GW280264X (*p* < 0.05 versus Model) (Figure 5E, right), consistent with the bioinformatic identification of RHBDF2 as a colitis-induced epithelial gene.

Western blot analysis of brain tissue corroborated these findings at the protein level (Figure 5F): Rhbdf2 protein abundance was elevated in the brains of Model mice compared with NC mice, and pharmacological ADAM17 blockade reduced cerebral Rhbdf2, Adam17 and Tnfα protein levels, indicating effective on-target inhibition of the ADAM17–TNFα inflammatory axis. Collectively, these in-vivo data demonstrate that UC-type intestinal inflammation is accompanied by RHBDF2 up-regulation and blood–brain barrier disruption, and that selective ADAM17 inhibition ameliorates both colitis severity and BBB damage, providing direct experimental support for the RHBDF2–ADAM17–TNFα axis as a mechanistic link between UC and BBBD.

## 4 Discussion

In this study, we combined bulk-tissue meta-analysis, cross-species orthologue mapping, single-cell transcriptomics, in-silico gene knockout, SMR-based genetic inference and an in-vivo colitis model to investigate the molecular link between UC and blood–brain barrier disruption. Several convergent findings emerged. First, four genes—TNF, RHBDF2, PILRA and CXCL16—were significantly and directionally concordantly up-regulated across five human UC cohorts and three mouse BBBD cohorts. Second, these genes form a protein–protein association module organized around the sheddase ADAM17, to which RHBDF2 is the essential activating chaperone and TNF and CXCL16 are canonical substrates. Third, RHBDF2 was predominantly up-regulated in colonic epithelial cells in UC, while at the human BBB its strongest inferred regulatory impact mapped to endothelial cells. Fourth, genetically predicted higher expression of RHBDF2 and PILRA was associated with increased risk of all-cause and Alzheimer’s dementia. Finally, in DSS-induced colitis, pharmacological blockade of ADAM17 with GW280264X attenuated intestinal inflammation, reduced cerebral Evans blue extravasation and lowered brain Rhbdf2, Adam17 and Tnfα protein levels. Taken together, these data are consistent with a model in which colitis-associated induction of the RHBDF2–ADAM17–TNFα axis contributes to blood–brain barrier injury.

A notable observation is the cellular compartmentalization of RHBDF2 in the gut. RHBDF2/iRhom2 has classically been studied in hematopoietic cells, where it governs ADAM17 maturation and stimulated TNFα release (Issuree et al., 2013; Maretzky et al., 2013), and iRhom2 is up-regulated in the colonic mucosa of patients with IBD and in experimental colitis, with deficiency ameliorating TNBS-induced disease (Kim et al., 2020). Our single-cell analysis adds spatial resolution to this picture: in the UC mucosa, RHBDF2 induction was most robust in the epithelial compartment, in line with the demonstration that inflammatory cytokines (TNFα plus interferon-γ) transcriptionally up-regulate iRhom2 in colon epithelial cells through JAK, NF-κB and AP-1 signaling, enhancing ADAM17 maturation and surface-molecule shedding (Giese et al., 2021). At the same time, our in-silico knockout experiment paints a more nuanced picture: virtual deletion of epithelial RHBDF2 de-repressed stress, apoptotic and inflammatory genes (including LGALS3, S100A10, B2M, IL6R and MCL1), implying that basal epithelial RHBDF2 also restrains stress and apoptotic programs. This apparent duality is consistent with the report that loss of Rhbdf2 on an Il10-deficient background precipitates spontaneous colitis (Geesala et al., 2019) and with the dual nature of ADAM17 itself, which is pro-inflammatory through TNF shedding yet protective of the intestinal barrier through EGFR-ligand release (Issuree et al., 2013; Brynskov et al., 2002). The UC-associated epithelial up-regulation of RHBDF2 may therefore reflect, at least in part, a compensatory response to epithelial injury, while its pro-inflammatory consequences are likely executed predominantly through ADAM17-mediated shedding in immune and endothelial compartments. Distinguishing these possibilities will require cell-type-specific perturbation *in vivo*. We also note that the bulk-tissue meta-analyses cannot themselves resolve cellular sources; the compartment assignments described above therefore rest on the single-cell data.

The network topology of the four shared genes lends mechanistic coherence to the cross-species intersection. Beyond TNF, CXCL16 is likewise an ADAM17-cleavable transmembrane chemokine; it is up-regulated in the serum and inflamed colon of patients with IBD, correlates with disease activity, and drives experimental colonic inflammation (Diegelmann et al., 2010; Uza et al., 2011; Kalliomäki et al., 2014). CXCL16 has also been implicated in CNS pathology, where its silencing attenuates neuroinflammation and M1 microglial polarization (Dingyi et al., 2024). The fourth shared gene, PILRA, encodes an inhibitory myeloid immunoreceptor whose variants modify Alzheimer’s disease risk and whose loss reshapes microglial immunometabolism and amyloid pathology (Weerakkody et al., 2025; Lopatko Lindman et al., 2022). That three of the four shared genes (TNF, CXCL16 and, upstream, RHBDF2) converge on a single proteolytic pathway, while PILRA provides an inhibitory counterweight on myeloid activation, suggests that the UC–BBBD intersection is not a random overlap of inflammatory genes but is structured around the RHBDF2–ADAM17 sheddase axis and its immunoregulatory balance. Nevertheless, the mouse BBBD cohorts model disparate insults (cerebrovascular injury with viral infection, cryptococcal meningoencephalitis and LPS-induced systemic inflammation) rather than colitis, and we cannot exclude that part of the shared signature reflects a generic innate-inflammatory program common to diverse brain injuries rather than UC-specific biology; BBB transcriptomes from colitis models will be required to resolve this.

At the blood–brain barrier itself, our in-silico knockout analysis assigned the dominant regulatory weight of RHBDF2 to endothelial cells—78 perturbed genes, compared with 14 in pericytes and 2 in astrocytes. The endothelial RHBDF2-regulated module is biologically telling: it contains ADAM17 and CXCL16 themselves, complement components (C1QA, C1QB, C1QC, C3), the microglial TYROBP–TREM2 signaling machinery, and vascular regulators including CAV1/CAV2, S1PR1 and the VEGFA–VEGFR2 pathway. Endothelial–pericyte crosstalk and complement activation are established drivers of BBB failure in dementia (Lin et al., 2021; Sweeney et al., 2019; Procter et al., 2021), and ADAM17 acts directly on brain endothelium by shedding glycocalyx and surface components (Zou et al., 2022; Yang et al., 2020; Wang et al., 2018). These observations support a model in which endothelial RHBDF2, induced in the setting of systemic intestinal inflammation, licenses ADAM17-dependent endothelial activation, complement engagement and leukocyte recruitment at the neurovascular unit. It should be emphasized, however, that these regulatory relationships are computational inferences from network rewiring inferred by scTenifoldKnk and do not substitute for experimental knockout. Moreover, the underlying dataset derives from peritumoral brain tissue of glioblastoma patients, which may exhibit tumor-related vascular abnormalities; although the paired within-patient design (sonicated versus non-sonicated samples from the same individual) mitigates this confounding, the cellular inferences at the human BBB should be extrapolated to non-tumor conditions with caution.

Among the SMR findings, the association between genetically predicted higher PILRA expression and Alzheimer’s dementia is the most robust, being the only association consistent with a shared causal variant (P_HEIDI = 0.094). The RHBDF2 associations with all-cause and Alzheimer’s dementia, although nominally significant after correction, showed HEIDI heterogeneity (P_HEIDI < 0.05) and therefore may reflect linkage rather than causality; we regard them as supportive but not definitive genetic evidence, and we consider the SMR results as a whole to be hypothesis-generating rather than definitive causal evidence. Notably, although PILRA showed the most robust genetic association, its role in Alzheimer’s disease is already well established (Weerakkody et al., 2025; Lopatko Lindman et al., 2022); because no prior study had linked RHBDF2 to blood–brain barrier biology, we prioritized the RHBDF2–ADAM17 axis for experimental validation on grounds of novelty and feasibility. The SMR analysis provides human genetic support for the disease relevance of two of the four shared genes: genetically predicted higher whole-blood expression of RHBDF2 and PILRA was associated with increased risk of all-cause and Alzheimer’s dementia, in a direction consistent with their up-regulation in BBBD transcriptomes. These results are in keeping with prior Mendelian randomization studies linking brain and blood gene expression to IBD and dementia (Liu et al., 2022; Li et al., 2025). Several caveats temper interpretation. First, only the PILRA–Alzheimer’s dementia association passed the HEIDI test (P_HEIDI = 0.094); the remaining significant associations, including those of RHBDF2, showed evidence of heterogeneity (P_HEIDI < 0.05) and may therefore reflect linkage rather than a shared causal variant. Second, each gene was instrumented by a single top cis-eQTL, precluding pleiotropy-robust sensitivity analyses. Third, whole-blood eQTLs are an imperfect proxy for expression regulation in brain barrier cell types. Finally, the direction of the PILRA association deserves caution: the AD-protective PILRA missense variant rs1859788 (G78R) is thought to reduce receptor function (Weerakkody et al., 2025; Lopatko Lindman et al., 2022), and a proteome-wide MR study estimated circulating PILRA to be protective against Alzheimer’s disease (Zhao et al., 2025), whereas our expression-based estimate associated higher PILRA transcript levels with increased risk. Expression level, protein abundance and receptor function need not be equivalent, and this discrepancy underscores that the SMR findings should be viewed as hypothesis-generating rather than definitive causal evidence. Two further caveats temper these genetic associations: they rest on single instruments, and the instruments derive from whole-blood cis-eQTLs, which are imperfect proxies for gene regulation in colon epithelium or brain endothelium; confirmation with tissue-matched eQTL data and formal colocalization analyses will be required.

Our in-vivo experiment provides the most direct support for the proposed axis. Consistent with classic demonstrations that experimental colitis increases BBB permeability to small molecules (Hathaway et al., 1999; Natah et al., 2005), DSS-treated mice showed marked cerebral Evans blue accumulation, and this leakage was significantly reduced by GW280264X (administered from the onset of DSS exposure, i.e., a preventive rather than therapeutic regimen) alongside improvements in body weight, disease activity, colon length and colonic cytokine expression. Whether ADAM17 inhibition is equally effective when administered after colitis onset—a therapeutic rather than preventive regimen—remains to be tested. These findings agree with in-vitro evidence that TNFα directly increases human BBB permeability (da Rocha et al., 2022), with the participation of ADAM17 in brain microvascular endothelial dysfunction (Zou et al., 2022; Yang et al., 2020; Wang et al., 2018), and with the reported anti-inflammatory efficacy of GW280264X in other models (Huner Yigit et al., 2025; Yu et al., 2025). An unexpected observation was that ADAM17 inhibition also lowered cerebral Rhbdf2 (and colonic Rhbdf2 mRNA): given that iRhom2 transcription is itself induced by inflammatory signaling through NF-κB and AP-1 (Giese et al., 2021), this raises the possibility of a positive feedback loop in which ADAM17-dependent release of TNFα and other mediators sustains RHBDF2 induction. If confirmed, such a loop could explain why transient pharmacological blockade produces sustained suppression of both the protease and its chaperone. Several caveats apply to this experiment. The acute 7-day DSS model does not capture the chronic relapsing course of human UC, and barrier permeability was assessed with a single small-molecule tracer without behavioral or cognitive endpoints. Moreover, GW280264X inhibits ADAM17 pharmacologically rather than RHBDF2 genetically; definitive proof of the in-vivo function of RHBDF2 at the BBB will therefore require cell-type-specific Rhbdf2 knockout models (e.g., endothelial Cdh5-CreERT2; Rhbdf2^fl/fl or intestinal epithelial Villin-Cre; Rhbdf2^fl/fl mice), which we plan to establish in future work. Finally, the Western blot findings were not accompanied by densitometric quantification and are therefore interpreted as supportive evidence.

From a translational perspective, our results add a molecular candidate to the emerging concept of a gut–brain vascular axis (Carloni et al., 2021; Carloni and Rescigno, 2022) and to the proposed entity of “enterogenic dementia” (Alshammari et al., 2025). Epidemiological studies indicate that patients with IBD carry an increased risk of dementia (Zhang et al., 2021; Zhang et al., 2022; Rønnow Sand et al., 2022), and BBB breakdown is an early and potentially modifiable event in cognitive decline (Lin et al., 2021; Li et al., 2021; Hergert et al., 2024). If the RHBDF2–ADAM17–TNFα axis indeed couples intestinal inflammation to barrier disruption, it may represent a druggable node: tissue-restricted targeting of iRhom2 has been proposed as a strategy to inhibit TACE in immune cells while sparing its homeostatic EGFR functions (Issuree et al., 2013; Giese et al., 2021), which could in principle circumvent the toxicity concerns that have hindered systemic TACE inhibition. Such speculation must, however, await direct genetic evidence that RHBDF2 at the BBB—rather than in the inflamed gut or peripheral immune cells—mediates the cerebral phenotype. Relatedly, our experimental validation was performed in mice. Obtaining human brain tissue from UC patients in a standardized, ethically compliant manner is extremely difficult; although RHBDF2 up-regulation was consistently observed in human UC transcriptomic cohorts and human colonic single-cell data, protein-level validation in human specimens remains to be performed. As a feasible alternative, we plan to quantify key molecules of the RHBDF2–ADAM17–TNFα axis in the blood of UC patients with different blood–brain barrier statuses in a follow-up study.

This study has several limitations, which are discussed in the relevant sections above: the mouse BBBD cohorts model heterogeneous non-colitic insults, and the shared signature may partly reflect generic neuroinflammation; the bulk meta-analyses cannot resolve cellular sources; the virtual-knockout analyses are computational inferences, and the human BBB single-cell dataset derives from peritumoral tissue; the SMR associations rest on single whole-blood instruments and, with one exception, did not pass the HEIDI test; and the in-vivo validation used an acute, preventive, pharmacological regimen without behavioral endpoints or densitometric quantification. Addressing these limitations—in particular through cell-type-specific Rhbdf2 knockout models, therapeutic-regimen studies and validation in human specimens—will define our future work.

## 5 Conclusion

Integrative multi-cohort meta-analysis, cross-species orthologue mapping, single-cell transcriptomics, in-silico gene knockout and SMR-based genetic inference converge on RHBDF2 as a shared, cell-type-resolved node linking ulcerative colitis to blood–brain barrier disruption, embedded in an ADAM17-centered protein module together with TNF, CXCL16 and PILRA. In DSS-induced colitis, pharmacological inhibition of ADAM17 with GW280264X attenuated intestinal inflammation, reduced cerebral Evans blue extravasation and lowered brain Rhbdf2, Adam17 and Tnfα protein levels, providing experimental support for the RHBDF2–ADAM17–TNFα axis as a mechanistic bridge between intestinal inflammation and blood–brain barrier injury. These findings nominate this axis as a candidate therapeutic target for the central nervous system complications of UC, while underscoring the need for cell-type-specific genetic perturbation of Rhbdf2, chronic colitis models and cognitive readouts to firmly establish causality.

## Data Availability

The publicly available datasets analyzed in this study can be found in the Gene Expression Omnibus (GEO; https://www.ncbi.nlm.nih.gov/geo/) under the accession numbers GSE193677, GSE206285, GSE224758, GSE235236 and GSE243625 (human ulcerative colitis), GSE172102, GSE230212 and GSE248275 (mouse blood–brain barrier disruption), GSE214695 (human ulcerative colitis single-cell RNA sequencing) and GSE208074 (human brain single-cell RNA sequencing). Whole-blood cis-eQTL summary statistics were obtained from the eQTLGen Consortium (https://www.eqtlgen.org/), and GWAS summary statistics for dementia endpoints were obtained from the FinnGen study (https://www.finngen.fi/). Other data supporting the findings of this study are available from the corresponding author upon reasonable request.

## Footnotes

1
https://www.ncbi.nlm.nih.gov/geo/

2
https://www.eqtlgen.org/

3
https://www.finngen.fi/

4
https://metascape.org/

5
https://string-db.org/

6
https://genemania.org/

7
https://www.proteinatlas.org/
