# Supplementary material for: Multi-cohort meta-analysis, single-cell and Mendelian randomization analyses with experimental validation identify RHBDF2 as a shared mediator of ulcerative colitis and blood–brain barrier disruption
Source: Front Synaptic Neurosci. 2026 Oct 7;18:1970578. doi: 10.3389/fnsyn.2026.1970578 (PMC13683702; doi:10.3389/fnsyn.2026.1970578)
Supplement: Supplementary file 1 [file Table_1.docx]

**Supplementary Table 1. Primer sequences used for RT-qPCR (mouse).**

| Gene | Forward primer (5′→3′) | Reverse primer (5′→3′) |
| --- | --- | --- |
| Gapdh | TTCACCACCATGGAGAAGGC | GGCATGGACTGTGGTCATGA |
| Il1b | GTTCAGGACAGCGACTTCTGGA | GAAGGAGAACTCCGCTGACTCT |
| Il6 | GAGTCCTTCAGAGAGATACAGAAAC | TGGTCTTGGTCCTTAGCCAC |
| Tnf | GATCGGTCCCCAAAGGGATG | GGTGGTTTGTGAGTGTGAGGG |
| Rhbdf2 | AGCCCTCATCCTCGTGTC | CTGGTCTAGCTCGTACTTCTCA |
